# The Complete Mitochondrial Genome of the Stingless Bee *Meliplebeia beccarii* (Hymenoptera: Apidae: Meliponini) and Insights into Unusual Gene Rearrangement

**DOI:** 10.3390/ijms262110588

**Published:** 2025-10-30

**Authors:** Shi-Jie Wang, Jiao Wu, Abebe Jenberie Wubie, Cheng-Ye Wang

**Affiliations:** 1 Environment and Plant Protection Institute, Chinese Academy of Tropical Agricultural Sciences, Haikou 571101, China; wang_yujie@yeah.net (S.-J.W.); jiaowu_1202@163.com (J.W.); 2 ORDA Ethiopia, Bahir Dar P.O. Box 132, Ethiopia; 3 Institute of Highland Forest Science, Chinese Academy of Forestry, Kunming 650224, China; 4 Key Laboratory of Breeding and Utilization of Resource Insects, National Forestry and Grassland Administration, Kunming 650224, China

**Keywords:** *Meliplebeia beccarii*, mitogenome, stingless bee, phylogenomics, gene rearrangement

## Abstract

The stingless bee *Meliplebeia beccarii*, endemic to Ethiopia, plays a crucial ecological and economic role through pollination and high-quality honey production. However, habitat degradation and anthropogenic pressures threaten its survival. In this study, we present the complete mitochondrial genome (mitogenome) of *M. beccarii*, revealing a compact structure of 15,458 bp with 13 protein-coding genes (PCGs), 19 tRNAs, and two rRNAs, characterized by an A + T bias (83.9%). Unique features include the absence of *trnI*, *trnK*, and *trnA*, translocation of *trnQ* and a novel inversion in the *trnT*-*trnP* combination. These findings highlight species-specific genomic adaptations. Phylogenetic analysis based on concatenated PCGs places *M. beccarii* within the Apidae lineage, contributing to a deeper understanding of stingless bee evolution. Our results underscore the utility of mitogenomic studies in biodiversity conservation and evolutionary biology, providing foundational insights for the management and preservation of *M. beccarii*.

## 1. Introduction

Stingless bees (Hymenoptera: Meliponini) are eusocial insects that construct and inhabit perennial nests and play a key role in tropical ecosystems [1,2]. Despite their small body size, they are highly efficient foragers that visit a wide variety range of tropical flowering plants and thus function as generalist pollinators [3]. In addition to their ecological services, stingless bees produce honey and propolis of considerable economic and pharmacological importance [4].

To date, approximately 600 species across 61 genera have been described worldwide [5]. The high species diversity increases the likelihood of cryptic taxa—distinct evolutionary lineages that are morphologically indistinguishable—and consequently complicates species delimitation. Traditional identification based on detailed morphometrics often fails to discriminate closely related taxa because diagnostic measurements commonly overlap, underscoring the need for integrative taxonomic approaches to achieve robust species boundaries. Finally, recent literature highlights a pervasive bias in pollination research toward managed honey bees, resulting in underrepresentation of wild bee taxa—including Meliponini—in ecological and applied studies [6]. Addressing both taxonomic uncertainty and the research bias are therefore essential for accurately assessing stingless bee diversity and their ecological and economic contributions.

*Meliplebeia beccarii*, a unique species known for its perennial colonies, construct their nests underground, where they reproduce and store honey and pollen [7]. *M. beccarii* is very docile, and its non-stinging nature makes them easy to manage and adapt around home gardens, offering both quality honey production and pollination services [7]. The honey produced by *M. beccarii* is a valuable bee product in Ethiopia, with a long tradition of consumption and several attributed traditional medicinal uses [7]. Despite its importance, African stingless bees remain comparatively understudied [8], limiting regionally relevant inferences about diversity, biogeography and ecosystem function. More specifically, stingless bee production, in Ethiopia, is suffering from habitat fragmentation, misuse of agrochemicals, prevalence of stingless bee enemies, lack of improved technologies for Meliponiculture, and lack of government attention are threats to this sector [9].

Mitochondrial genome (mitogenome) data have been used in phylogenetics and population genetics studies of many insect groups [10] particularly useful for species conservation and biodiversity assessments [11]. Mitogenomic data have recently clarified evolutionary relationships in social bees and improved resolution where single-gene markers proved insufficient [12,13]. In previous studies, we have characterized the mitogenomes of several Meliponini species [14,15,16], highlighting the importance of these sequences for understanding stingless bees. This understanding is crucial for developing effective management and conservation strategies. Despite these recent studies, complete mitogenomes remain sparse for native stingless bee species in Ethiopia, which constrains robust phylogenetic placement and the development of mitochondrial reference libraries that underpin species delimitation and mitochondrial-based biodiversity surveys. In the present study, we sequenced the complete mitogenome of *M. beccarii* and analyzed the gene order, nucleotide composition, and secondary structures of its tRNA genes. Additionally, we reconstructed the phylogenetic relationships between *M. beccarii* and other insects in Apoidea, contributing to broader research on mitochondrial function and its role in ecological adaptation.

## 2. Results and Discussion

### 2.1. General Mitogenome Features and Nucleotide Composition

**Figure 1:**
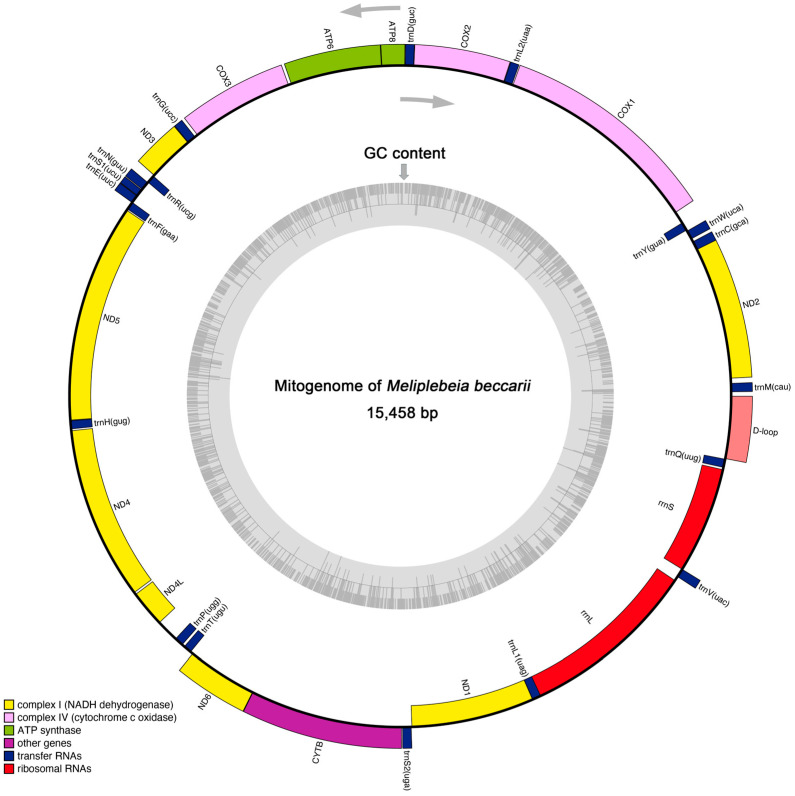
The circular map of complete mitogenome of *M. beccarii*. Genes distributed on the inner and outer sides of the loop exhibit distinct transcriptional orientations, as indicated by the two directional arrows. These orientations correspond to genes located on the mitochondrial genome’s light (N) strand and heavy (J) strand, respectively. The inner gray ring indicates the GC content across the entire mitogenome, analyzed using a sliding window approach.

The insect mitogenome is typically a circular, double-stranded molecule of 15–18 kb in length [10]. The mitogenome of *M. beccarii* was 15,458 bp in size and comprised 13 protein-coding genes (PCGs), 19 transfer RNA (tRNA) genes, two ribosomal RNA (rRNA) genes, and a noncoding control region (D-loop). These genes were distributed across both the J-strand (majority) and N-strand (Figure 1 and Table 1). However, compared to other insects [17], the number and distribution of genes in the *M. beccarii* mitogenome appear to be inconsistent. The presence of overlapping regions (e.g., *COX2* and *trnD*) and intergenic spacers (e.g., *trnH* and *ND4*) indicated a compact genome structure and characteristic of mitochondria. These regions may influence gene expression or genome stability. The varying start codons observed in mitochondrial PCGs suggested possible translational mechanisms specific to *M. beccarii* (Table 1). Additionally, the genes with long sequences, such as *COX1*, *ND5*, and *CYTB*, represented key components of the oxidative phosphorylation pathway, emphasizing their importance in mitochondrial function. The large D-loop region in the *M. beccarii* mitogenome suggests its potential regulatory role in replication and transcription, consistent with findings in other mitogenomes [18].

The base composition of the *M. beccarii* mitogenome was 41.5% for A, 7.1% for G, 42.4% for T, and 9.1% for C (Table 2). The A + T content was 83.9%, consistent with the high A + T bias typical of mitogenomes [15]. This bias was also evident in individual regions: PCGs (83.3%), tRNAs (87.6%), and rRNAs (80.7%) (Table 2). As expected for an A + T-rich genome, the percentages of A and T were higher than those of C and G. The AT-skew for the whole genome was slightly negative (−0.011), indicating a small excess of T over A, while the GC-skew was more strongly negative (−0.125), reflecting a higher proportion of C relative to G. Interestingly, specific regions, such as tRNAs and rRNAs, displayed positive AT-skew and GC-skew values, indicating regional variations in nucleotide composition. Among these, tRNAs exhibited the highest A + T content (87.6%) in the *M. beccarii* mitogenome. The sequences of *M. beccarii* and other species in Apidae displayed consistently high A + T content (see Figure S1, Supplementary Materials), particularly in mitochondrial control regions, potentially reflecting unique regulatory requirements or evolutionary adaptations.

### 2.2. PCGs and Codon Usage Bias

The total length of the 13 PCGs in the *M. beccarii* mitogenome was 11,121 bp. Among these, 12 PCGs utilized the standard ATN as their start codon. For stop codons, 11 PCGs terminated with the common mitochondrial stop codon TAA, while *CYTB* used TAG as its stop codon (Table 1). Notably, the intergenic length for *COX2* was −2, indicating an overlap with adjacent genes.

**Figure 2:**
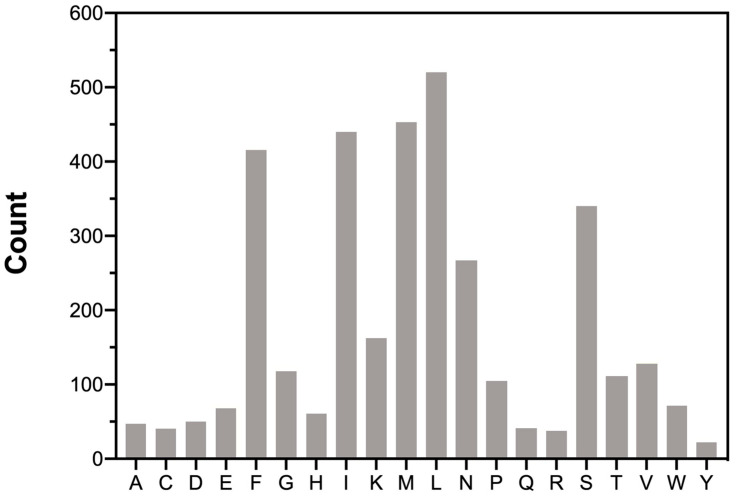
Amino acids distribution in the *M. beccarii* mitogenome. The number on the left is the total number of each amino acid. The 20 amino acid types are represented on the *X*-axis.

The total amino acids count and distribution provide insights into translational preferences and constraints within the mitogenome of *M. beccarii* (Figure 2). The codon usage in the mitochondrial PCGs was analyzed to determine the total codon count and relative synonymous codon usage (RSCU) for each codon (see Table S1, Supplementary Materials). Codon usage bias, often reflected in deviations of RSCU values, is associated with translational efficiency, gene expression levels, and evolutionary pressures. The predominance of A + T-rich codons, such as UUA (L) and AUA (M), aligns with the A + T bias of the *M. beccarii* mitogenome. UUA and AUA showed strong preference, with high RSCU values of 4.80 and 3.60, respectively, highlighting a notable codon usage bias. UUU, UAU, and UGA also exhibited strong bias, consistent with the A + T-rich nature of the genome.

**Figure 3:**
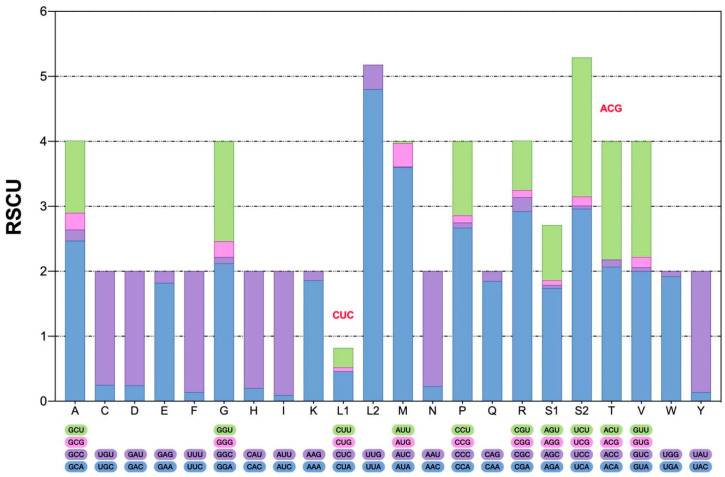
The relative synonymous codon usage (RSCU) in the mitogenome of *M. beccarii*. The type of codon is on the *X*-axis. Codons on bars (red font letters) indicate that they are not found in *M. beccarii*.

Conversely, certain codons, such as CGC (R) and ACG (T), were underrepresented, likely due to limited availability of corresponding tRNAs, which may influence protein synthesis efficiency. Rare codons, including CUG (L) and AUC (M), showed low or zero usage, with RSCU values near 0, indicating underrepresentation in the mitogenome. Among stop codons, UAA was predominant (RSCU = 1.85), while UAG was used sparingly (RSCU = 0.15). Interestingly, UGA, typically a stop codon, encoded tryptophan (W) in the mitochondria, with moderate usage (RSCU = 1.92). The absence of certain codons in *M. beccarii* may reflect evolutionary preferences in codon selection or functional loss (Figure 3).

### 2.3. Ribosomal and Transfer RNAs

The two rRNA genes located on the N-strand exhibited an A + T content of 80.7% (Table 1 and Table 2). The *rrnS* gene, 808 bp in length, had an A + T content of 79.8%, while the *rrnL* gene, 1354 bp in size, showed an A + T content of 81.3%. Conserved structural elements and motifs in these genes reflect evolutionary pressures to maintain ribosomal functionality across Apidae. Conversely, unique structural variations may signify species-specific adaptations or divergence in mitochondrial translation mechanisms. Notably, structural variations or conserved regions in the 16S rRNA may represent evolutionary adaptations of *M. beccarii* to its ecological niche (see Figure S2, Supplementary Materials). Similarly, deviations from the canonical 12S rRNA secondary structure could indicate unique evolutionary or functional adaptations specific to *M. beccarii* (see Figure S3, Supplementary Materials).

tRNAs play a fundamental role in protein synthesis, serving as adaptors that translate mRNA codons into amino acids. In mitogenomes, tRNAs frequently exhibit unique structural adaptations driven by the compactness and evolutionary pressure characteristic of these genomes. Among the 19 tRNA genes distributed across the *M. beccarii* mitogenome, 11 genes were located on the J-strand, while the remaining eight genes were situated on the N-strand (Table 1). The secondary structures of mitochondrial tRNAs (see Figure S4, Supplementary Materials) revealed that all tRNAs can be folded into typical cloverleaf secondary structures. However, mitochondrial tRNAs often deviate from these canonical structures, and such deviations were observed in *M. beccarii*. These variations, shaped by relaxed selection pressures and functional adaptations, may highlight structural features and biological significance unique to the species. For *M. beccarii*, these structural characteristics likely reflect adaptations to its ecological niche and metabolic demands.

### 2.4. Phylogenetic Analysis

**Figure 4:**
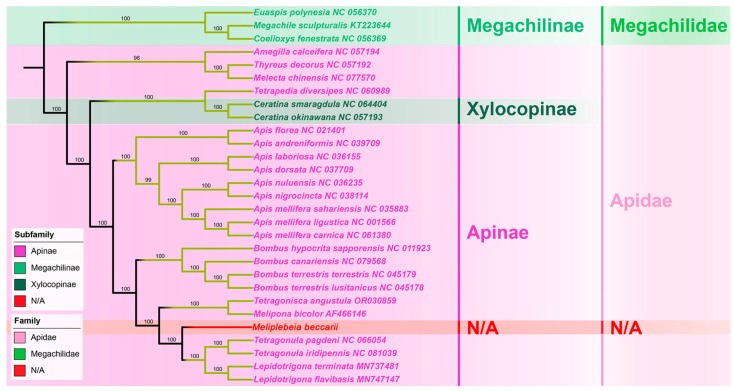
Phylogenetic trees of 29 Apoidea species (Apidae + Megachilidae) based on mitochondrial 13 PCGs data, inferred by Maximum likelihood method. Values on each node indicate the bootstrap percentages. The GenBank accession numbers of each sequence were provided after the species name.

The whole mitogenome sequence is a better choice for a phylogenetic analysis than partial mitochondrial sequences or a combination of partial mitochondrial sequences and a few nuclear genes, however, the phylogenetic relationships between stingless bees and other hymenopteran insects have not been analyzed based on complete mitogenomes [15]. In this study, *M. beccarii* was clustered with other 28 representative hymenopteran species whose complete mitogenomes have been reported. In the phylogenetic tree, mitogenome sequences of all stingless bees (Meliponini) + bumble bees (Bombini), formed a monophyletic clade. The phylogenetic analysis revealed evolutionary relationships among representative Apoidea species, placing *M. beccarii* within its monophyletic clade and showing its closest evolutionary affinity to the sister group comprising *Tetragonula* and *Lepidotrigona* species. (Figure 4). Analysis based on 13 PCGs provided a robust framework for studying evolutionary relationships, as these genes are conserved and informative across diverse taxa. The clustering of species in the tree reflects shared evolutionary ancestry, with closer nodes indicating more recent divergence events. High bootstrap values at most nodes suggested strong support for the inferred relationships. This relationship is consistent with the phylogeny reconstructed with a multigene approach based on nuclear, mitochondrial, and ribosomal loci [19]. The families Apidae, represented in this study by the species of Meliponini, Bombus, and Apis, formed a monophyletic clade, as previously suggested by molecular sequence data [14,15,16].

### 2.5. Gene Order and Rearrangement

**Figure 5:**
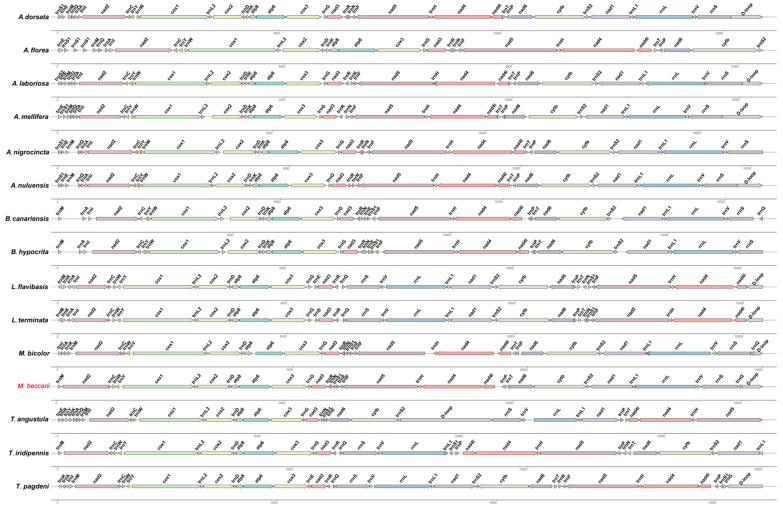
Comparative mitochondrial gene-order map of *M. beccarii* and 14 other Apidae species. Arrows of different colors indicate orthologous genes, with their direction representing transcriptional orientation. Identical genes across mitogenomes are marked with identical colors. Arrow lengths are proportional to the lengths of the corresponding genes. GenBank accession numbers for all mitogenomes are listed in the Data Availability Statement.

The gene order of the mitogenome of *M. beccarii* was compared with that of 14 species in Apidae (including *Apis*, *Bombus*, Meliponini), providing insights into the conservation and variability of mitogenome organization across the subfamily (Figure 5). The results showed that gene orders were largely preserved across multiple species, supporting their phylogenetic relationships. However, the mitogenome of *M. beccarii* exhibited unique features and rearrangements compared to other species, indicating species-specific adaptations or evolutionary divergence within Apidae.

In contrast to the highly conserved gene orders typically observed in most invertebrate mitogenomes [20], significant rearrangements were identified in the stingless bee mitogenomes. Notably, in *M. beccarii*, the positions of several rRNA and tRNA genes were dramatically changed (Figure 5). The absence of *trnI*, *trnK*, and *trnA* in the *M. beccarii* mitogenome may be compensated by functional replacements or alternative genetic mechanisms. Nuclear-encoded counterparts are likely replacing mitochondrial tRNAs even in systems with recent mitochondrial tRNA gene loss, and the redundant import of a nuclear-encoded tRNA may provide a mechanism for functional replacement between translation systems separated by billions of years of evolutionary divergence [21].

Additionally, *trnQ* of *M. beccarii* mitogenome translocated and inserted between rrnS and *D-loop*, and this event was shared with *M. bicolor*, indicating their close affinity from another perspective. The direction of *trnT* was inverted in the *M. beccarii* mitogenome compared to other *Apis* species. Meanwhile, a switch of the location of *trnT* and *trnP* occurred between *M. beccarii* and *M. bicolor*, and this unusual gene rearrangement occurred between two closely related species might hint a recent functional adaptation (Figure 5). Comparative analyses reveal high rates of gene rearrangement and notable differences in tRNA complements among Meliponini, Bombini and Apini, suggesting lineage-specific mitogenome dynamics that may underlie observed tRNA losses or atypical annotations [12]. tRNA rearrangements are common across all three tribes of social bees [12], for example high gene rearrangement in *Lepidotrigona* mitogenomes [15]. These rearrangements may reflect the evolutionary dynamics and rates specific to certain species or genera [22], which are not necessarily constrained by natural selection [23]. tRNA loss and gene rearrangement seen in *M. beccarii* are not isolated phenomena but part of a broader pattern of mitogenomic plasticity within the tribe. For instance, *L. terminata* and *L. flavibasis* both possess typical 22 tRNAs, and the two *Lepidotrigona* mitogenomes share highly rearranged but mutually similar gene orders [14,15]. *Melipona bicolor* carries a conserved tRNA translocation event across Meliponini (i.e., distinctive, lineage-specific tRNA rearrangements rather than tRNA loss) [24]. These comparisons indicate that Mitogenome architecture within Meliponini is highly labile, making Meliponini a hotspot of mitogenome structural evolution [12].

Mitochondrial gene rearrangements in vertebrates generally align with the duplication/random loss model [25,26]. However, many invertebrate mitochondrial rearrangements deviate from this mechanism [27,28,29,30]. Among invertebrates, most rearrangements involve tRNA genes and are thought to have occurred during the evolutionary history of Hymenoptera. Future research will aim to uncover the molecular evolutionary trends underlying these rearrangements in stingless bees.

## 3. Materials and Methods

### 3.1. Stingless Bee Acquisition and DNA Extraction

Individuals of *M. beccarii* were obtained from Hulet Eju Enesie (Motta) district, Amhara region, Ethiopia (37.5242° E, 11.0609° N). After collecting, the fresh materials were preserved in 100% ethanol immediately and stored in −80 °C refrigerator before DNA extraction. The total genomic DNA was extracted from the chest muscles of the specimens with a Tissue DNA Kit (TIANGEN Biotech, Beijing, China) following the manufacturer’s instructions. The total DNA content was detected by using a Qubit dsDNA HS assay kit (Invitrogen, Carlsbad, CA, USA).

### 3.2. Sequencing and Assembly

The library was constructed with the 1.0 μg genomic DNA by using KAPA Hyper Prep Kits (KAPA Biosystems, Wilmington, MA, USA). The sequencing work of the complete *M. beccarii* mitogenome was performed by an Illumina Nextseq500 by using the Next Generation Sequencing technology to obtain the original file. After removing the connector and the unmatched, short, and poor-quality reads, the remaining high-quality reads were assembled from scratch using IDBA-UD and SPAdes [31]. After the mitogenome was generated by de novo assembly, PCGs of the *M. beccarii* mitogenome were identified using BLAST (+2.16.0 version) search against the nucleotide databases in NCBI [https://www.ncbi.nlm.nih.gov] (accessed on 20 January 2025), and the tRNA genes were identified using the tRNAscan-SE search server [32]. The final assembled mitogenome was verified, and the secondary structures of the tRNAs were predicted on the MITOS web server [33]. The mitogenome map was generated by the molecular biology tool CGView [34].

### 3.3. Phylogeny Analysis

To compare the *M. beccarii* mitogenome with other hymenopteran insects, concatenated PCGs sequences from mitogenomes were aligned through ClustalW in the Mega 7.0 software package [35]. The mitogenomes adopted in this analysis were downloaded from GenBank, and the accession number for each mitogenome is provided alongside the species name in the phylogenetic tree. The maximum likelihood method was used to construct phylogenetic relationships with default settings, and the bootstrap values were estimated using 500 replicates. The base composition and relative synonymous codon usage (RSCU) were also analyzed using the Mega 7.0 software package [35] and PhyloSuite software package (v1.2.3) [36]. The GC skew was computed according to the following formula: GC skew = [G − C]/[G + C] [37].

### 3.4. Gene Rearrangement Evaluation

The related mitogenomes were downloaded from GenBank, and the gene orders were extracted and potential rearrangement of each mitogenome was calculated with PhyloSuite [35] and visualized on the iTOL website server [38]. The accession numbers of all the mitogenomes analyzed in this study could be found in the Data Availability Statement.

## 4. Conclusions

We reported the first complete mitochondrial genome of the Ethiopian endemic bee *M. beccarii* (15,458 bp), and the absence of *trnI*, *trnK*, and *trnA*, translocation of *trnQ* and a novel inversion in the *trnT-trnP* combination may have profound implications for mitogenome function and evolution. Phylogenetic analysis based on the 13 PCGs places *M. beccarii* within Apidae and supports the utility of mitogenomic data for resolving Meliponini relationships. These results provide a genomic baseline for future studies of population genetics, comparative mitogenomics, and conservation planning for *M. beccarii*. Further work should expand sampling across populations to assess intraspecific variation, employ complementary nuclear markers to corroborate phylogenetic inferences, and investigate the functional consequences of the observed gene rearrangements.

## Figures and Tables

**Table 1:** Mitogenome composition of *M. beccarii*.

Gene	Strand	Nucleotide Number	Intergenic Length	Start Codon	Stop Codon	Anticodon
*trnM*	J	30–96	34			CAU
*ND2*	J	131–1147	−20	ATT	TAA	
*trnC*	J	1128–1197	20			GCA
*trnW*	J	1218–1287	4			UCA
*trnY*	N	1292–1358	88			GUA
*COX1*	J	1447–3012	4	ATC	TAA	
*trnL2*	J	3017–3081	0			UAA
*COX2*	J	3082–3765	−2			
*trnD*	J	3764–3829	0			GUC
*ATP8*	J	3830–4003	−1	ATT	TAA	
*ATP6*	J	4003–4692	18	ATA	TAA	
*COX3*	J	4711–5490	19	ATG	TAA	
*trnG*	J	5510–5577	0			UCC
*ND3*	J	5578–5931	4	ATA	TAA	
*trnR*	N	5936–6000	−1			UCG
*trnN*	J	6000–6068	2			GUU
*trnS1*	J	6071–6127	0			UCU
*trnE*	J	6128–6192	−6			UUC
*trnF*	N	6187–6251	6			GAA
*ND5*	N	6258–7910	65	ATT	TAA	
*trnH*	N	7976–7911	74			GUG
*ND4*	N	7986–9296	13	ATA	TAA	
*ND4l*	N	9310–9588	166	ATA	TAA	
*trnP*	N	9755–9824	17			UGG
*trnT*	N	9842–9906	16			UGU
*ND6*	J	9923–10,456	−1	ATG	TAA	
*CYTB*	J	10,456–11,604	4	ATG	TAG	
*trnS2*	J	11,609–11,676	1			UGA
*ND1*	N	11,678–12,607	−3	ATA	TAA	
*trnL1*	N	12,605–12,673	−23			UAG
*rrnL*	N	12,651–14,004	30			
*trnV*	J	14,035–14,103	−4			UAC
*rrnS*	N	14,100–14,907	13			
*trnQ*	N	14,921–14,987	0			UUG
*D-loop*	-	14,988–15,458	0			

**Table 2:** Base composition and skewness of the *M. beccarii* mitogenome ^1^.

	Length (bp)	A (bp)	C (bp)	G (bp)	T (bp)	A (%)	C (%)	G (%)	T (%)	A + T (%)	AT-Skew	GC-Skew
Whole genome	15,458	6408	1405	1092	6553	41.5	9.1	7.1	42.4	83.9	−0.011	−0.125
PCGs ^2^	11,121	4538	1012	843	4728	40.8	9.1	7.6	42.5	83.3	−0.020	−0.091
tRNAs	1268	570	66	90	542	44.9	5.2	7.1	42.7	87.6	0.025	0.154
rRNAs	2162	876	160	256	870	40.5	7.4	11.8	40.2	80.7	0.003	0.231
*rrnL* (N-strand)	1354	542	103	150	559	40.0	7.6	11.1	41.3	81.3	−0.015	0.186
*rrnS* (N-strand)	808	334	57	106	311	41.3	7.1	13.1	38.5	79.8	0.036	0.301

^1^ The parameters in the table are calculated using J-chain (Forward direction) except *rrnL* and *rrnS* which are calculated using N-chain (Reverse direction). ^2^ PCG means protein coding gene.

## Data Availability

All data are included in this paper. Complete mitochondrial genomes assembled and annotated in the present study are available on the NCBI GenBank Database. Accession numbers are as follow: *Apis andreniformis* [NC039709], *Apis dorsata* [NC037709], *Apis florea* [NC021401], *Apis laboriosa* [NC036155], *Apis mellifera carnica* [NC061380], *Apis mellifera ligustica* [NC001566], *Apis mellifera sahariensis* [NC035833], *Apis nigrocincta* [NC038114], *Apis nuluensis* [NC036235], *Bombus canariensis* [NC079568], *Bombus hypocrita sapporensis* [NC011923], *Bombus terrestris lusitanicus* [NC045178], *Bombus terrestris terrestris* [NC045179], *Lepidotrigona flavibasis* [MN747147], *Lepidotrigona terminata* [MN737481], *Melipona bicolor* [AF466146], *Tetragonisca angustula* [OR030859], *Tetragonula iridipennis* [NC081039], *Tetragonula pagdeni* [NC066054], *Ceratina okinawana* [NC057193], *Ceratina smaragdula* [NC064404], *Tetrapedia diversipes* [NC060989], *Melecta chinensis* [NC077570], *Thyreus decorus* [NC057192], *Amegilla calceifera* [NC057194], *Coelioxys fenestrate* [NC056369], *Megachile sculpturalis* [KT223644], *Euaspis Polynesia* [NC056370].

## Supplementary Materials

The following supporting information can be downloaded at: https://www.mdpi.com/article/10.3390/ijms262110588/s1.
