# Supplementary material for: The Complete Mitochondrial Genome of the Stingless Bee Meliplebeia beccarii (Hymenoptera: Apidae: Meliponini) and Insights into Unusual Gene Rearrangement
Source: Int J Mol Sci. 2025 Oct 30;26(21):10588. doi: 10.3390/ijms262110588 (PMC12610537; doi:10.3390/ijms262110588)
Supplement: Supplementary file 1 [file ijms-26-10588-s001.zip › ijms-3908184-supplementary.pdf]

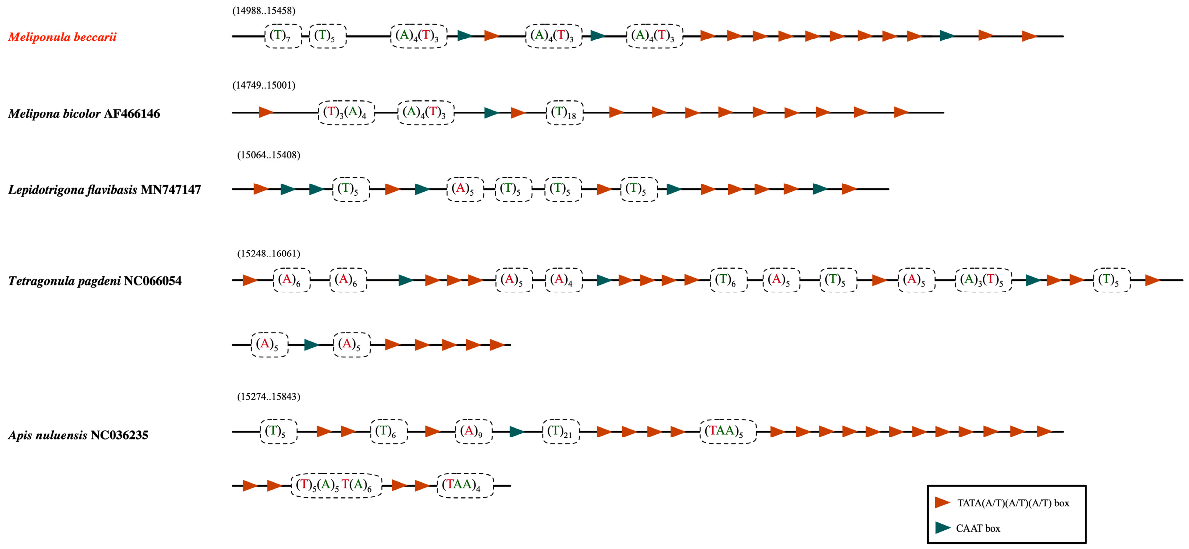

Figure S1: Structures of the A + T-rich regions of *M. beccarii* and other species in Apidae.

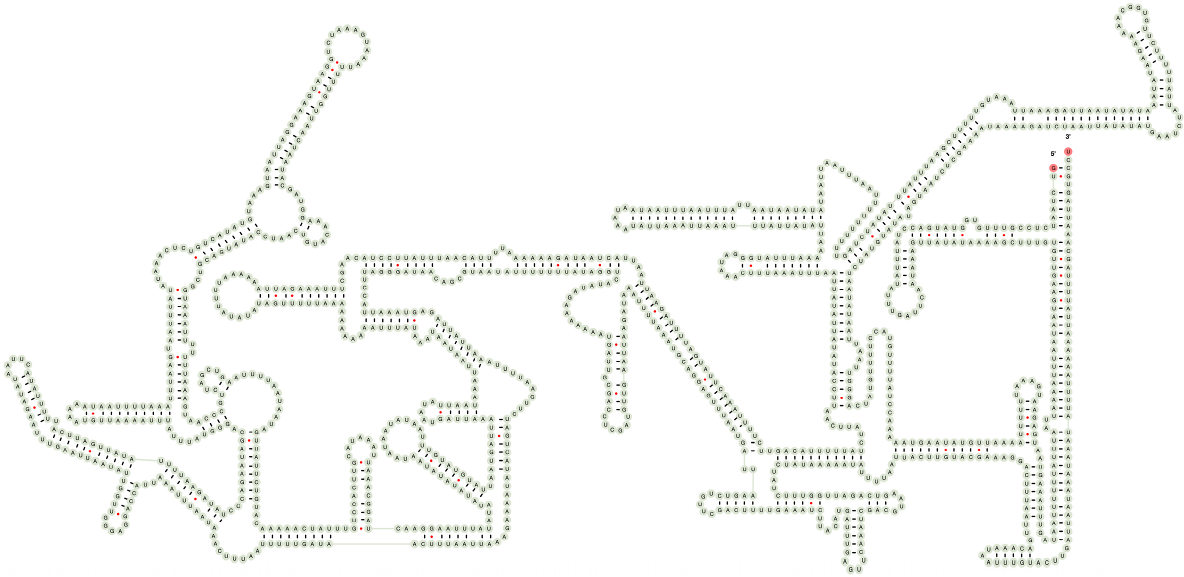

Figure S2: Predicted secondary structures of 16S in *M. beccarii* mitochondrial genome. '-' indicates Watson-Crick base pairing and '·' indicates GU base pairing.

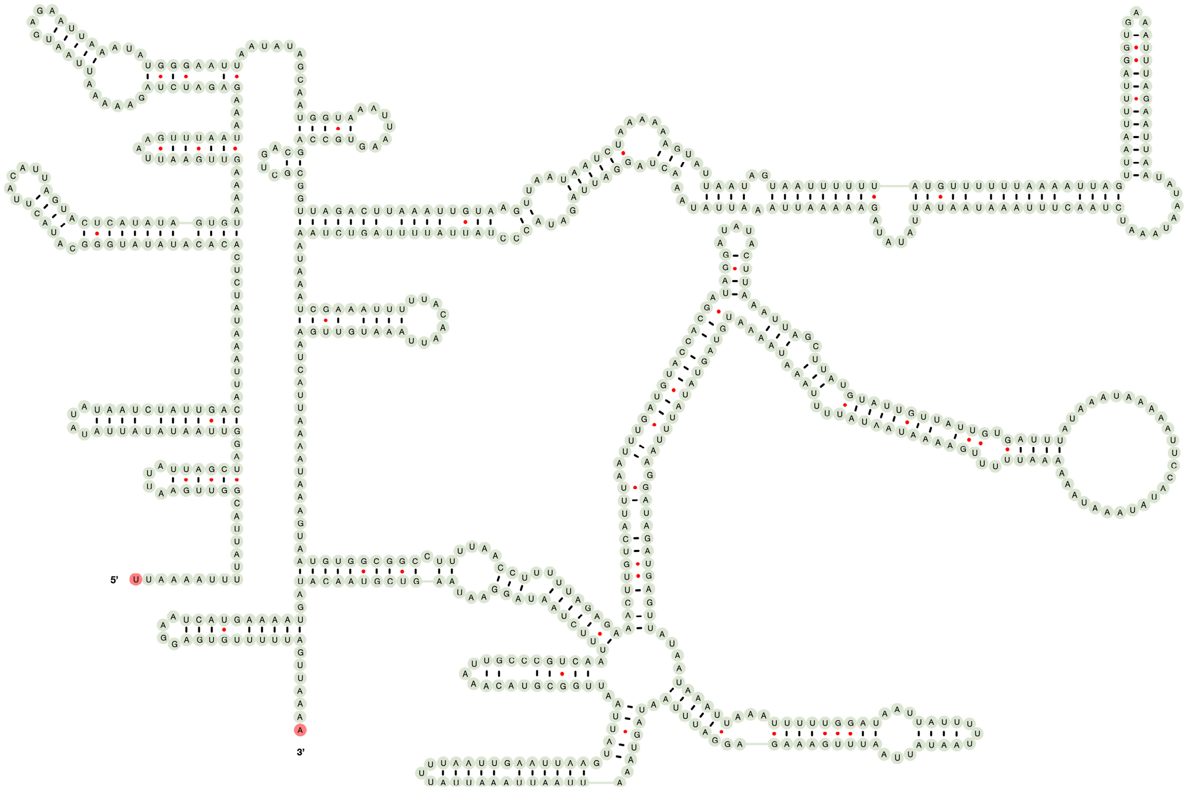

Figure S3: Predicted secondary structures of 12S in *M. beccarii* mitochondrial genome. '-' indicates Watson-Crick base pairing and '·' indicates GU base pairing.

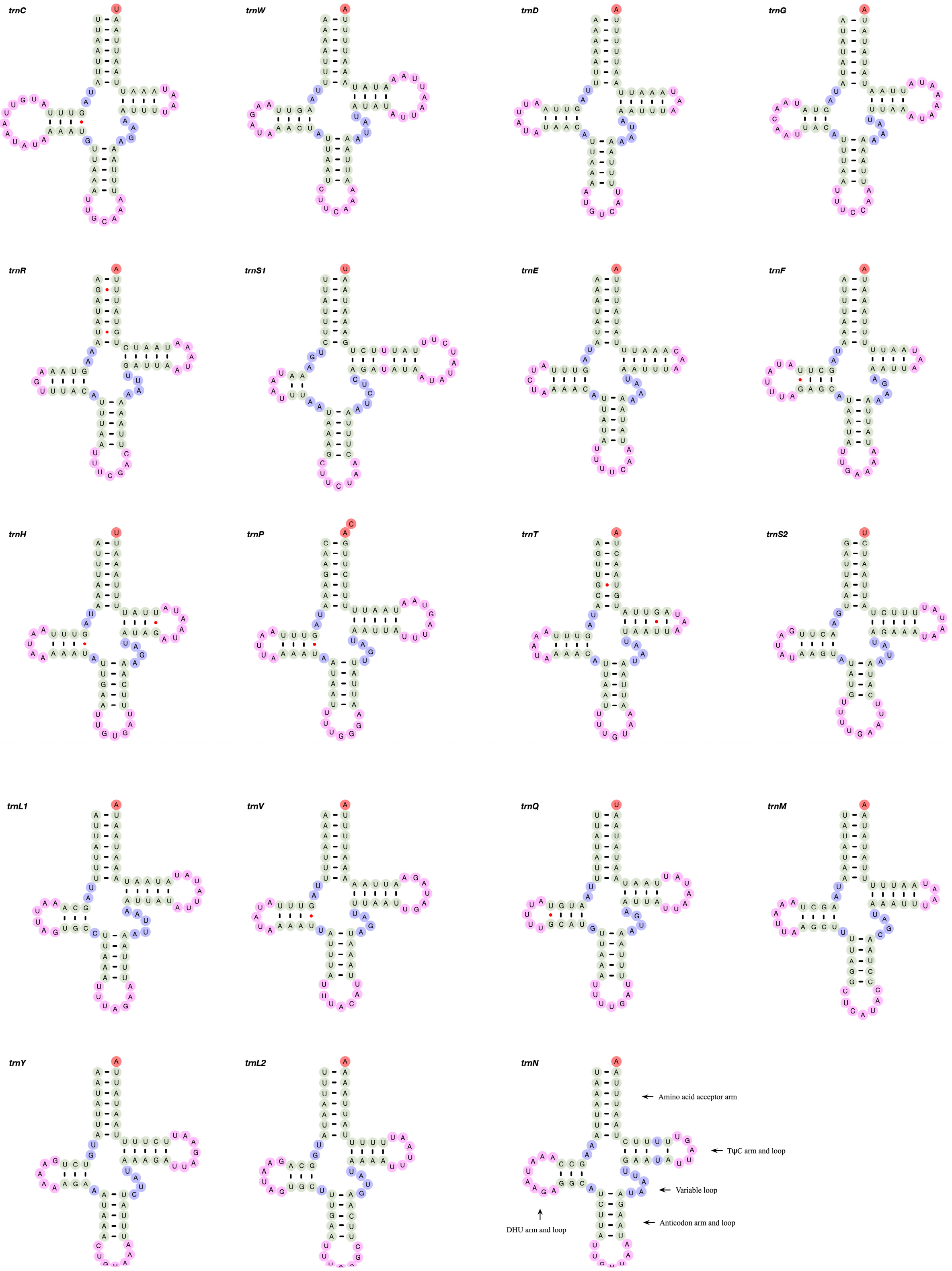

Figure S4: Predicted secondary structures of tRNAs in *M. beccarii* mitochondrial genome. '-' indicates Watson-Crick base pairing and '·' indicates GU base pairing.

Table S1. Codon usage of mitochondrial protein coding genes (PCGs) in *M. beccarii*.

Codon	Count	RSCU	Codon	Count	RSCU	Codon	Count	RSCU
GCA (A)	29	2.47	CUA (L)	40	0.46	AGA (S)	74	1.74
GCC (A)	2	0.17	CUC (L)	0	0.00	AGC (S)	2	0.05
GCG (A)	3	0.26	CUG (L)	5	0.06	AGG (S)	3	0.07
GCU (A)	13	1.11	CUU (L)	26	0.30	AGU (S)	36	0.85
UGC (C)	5	0.25	UUA (L)	415	4.80	UCA (S)	126	2.96
UGU (C)	35	1.75	UUG (L)	33	0.38	UCC (S)	2	0.05
GAC (D)	6	0.24	AUA (M)	407	3.60	UCG (S)	6	0.14
GAU (D)	44	1.76	AUC (M)	1	0.01	UCU (S)	91	2.14
GAA (E)	62	1.82	AUG (M)	41	0.36	ACA (T)	57	2.07
GAG (E)	6	0.18	AUU (M)	3	0.03	ACC (T)	3	0.11
UUC (F)	30	0.14	AAC (N)	31	0.23	ACG (T)	0	0.00
UUU (F)	385	1.86	AAU (N)	236	1.77	ACU (T)	50	1.82
GGA (G)	62	2.12	CCA (P)	70	2.67	GUA (V)	64	2.00
GGC (G)	3	0.10	CCC (P)	2	0.08	GUC (V)	2	0.06
GGG (G)	7	0.24	CCG (P)	3	0.11	GUG (V)	5	0.16
GGU (G)	45	1.54	CCU (P)	30	1.14	GUU (V)	57	1.78
CAC (H)	6	0.20	CAA (Q)	37	1.85	UGA (W)	68	1.92
CAU (H)	55	1.80	CAG (Q)	3	0.15	UGG (W)	3	0.08
AUC (I)	20	0.09	CGA (R)	27	2.92	UAC (Y)	16	0.14
AUU (I)	419	1.91	CGC (R)	2	0.22	UAU (Y)	210	1.86
AAA (K)	151	1.86	CGG (R)	1	0.11	UAA*	12	1.85
AAG (K)	11	0.14	CGU (R)	7	0.76	UAG*	1	0.15
